# Analysis of 777 cases with obstruction of the ureter or extrahepatic bile duct by ultrasonography after normal saline retention enema

**DOI:** 10.1186/2036-7902-4-6

**Published:** 2012-04-17

**Authors:** Chong Tang, Xuegang Wu, Qiuhong Fan, Zhensheng Deng

**Affiliations:** 1 Department of Ultrasonics of Xi'an Electric Central Hospital, Xi'an, 710032, Shaanxi Province, China; 2 Institute of Biomedical Engineering of Central South University, #932 Lushan Nanlu Road Changsha, 410083, Hunan Province, China

**Keywords:** ultrasound, ureteral obstruction, extrahepatic bile duct obstruction, normal saline retention enema

## Abstract

**Background:**

Conventional transabdominal ultrasound usually fails to visualize parts of the ureter or extrahepatic bile duct covered by bowel gas. In this study, we propose a new method for gaining acoustic access to the ureters and extrahepatic bile duct to help determine the nature of obstruction to these structures when conventional transabdominal ultrasound fails.

**Methods:**

The normal saline retention enema method, that is, using normal saline-filled colons to gain acoustic access to the bilateral ureters and extrahepatic bile duct and detecting the lesions with transabdominal ultrasonic diagnostic apparatus, was applied to 777 patients with obstructive lesions, including 603 with hydroureter and 174 with dilated common bile duct, which were not visualized by conventional ultrasonography. The follow-up data of all the patients were collected to verify the results obtained by this method.

**Results:**

Of the 755 patients who successfully finished the examination after normal saline retention enema (the success rate of the enema is about 98%), the nature of obstruction in 718 patients was determined (the visualizing rate is approximately 95%), including 533 with ureteral calculus, 23 with ureteral stricture, 129 with extrahepatic bile duct calculus, and 33 with common bile duct tumor.

**Conclusions:**

Colons filled fully with normal saline can surely give acoustic access to the bilateral ureters and extrahepatic bile duct so as to determine the nature of obstruction of these structures when conventional transabdominal ultrasound fails.

## Background

Obstructive diseases of the ureters and extrahepatic bile duct caused by lithiasis, cancer, stricture, etc. are commonly encountered in the clinical setting, and their diagnosis mainly relies on imaging, such as X-ray contrast radiography, computed tomography (CT), magnetic resonance imaging (MRI), and ultrasonography. Though CT and MRI are more convenient in getting reliable diagnostic results, there are some disadvantages such as much higher cost and higher risks caused by the ionizing radiation of CT and the strong magnetic field of an MRI scanner [1,2]. However, ultrasound is commonly available, inexpensive to operate, and has no risk from radiation exposure or strong magnetic field, so it is commonly used as a diagnostic tool in patients with obstructive diseases of the urinary system or extrahepatic bile duct [3].

The disadvantage is that some obstruction of the ureter and extrahepatic bile duct is difficult or even impossible to be visualized due to the effect of bowel gas, the patient's physical type, equipment condition, and other factors [4-9]. To eliminate the effect of bowel gas in the examination of obstructive diseases of the bilateral ureters and extrahepatic biliary duct, the method of normal saline retention enema is employed in this study to help determine the nature of obstruction to these structures when conventional transabdominal ultrasound fails.

## Methods

### Principles

Anatomically, the ureters are nearly parallel to the sigmoid colon, descending colon, ascending colon, and ileocecus, and the extrahepatic bile duct is nearly overlapped by the transverse colon. Filling those colons with normal saline, we can gain acoustic access to the certain part of the bilateral ureters and the extrahepatic bile duct which needs to be examined so as to visualize the obstructive diseases of these structures by ultrasonography.

### Subjects

Since 1990, 777 cases with ages 14 to 76 years old (468 males and 309 females) have been accumulated and divided into group A and group B according to the location of their obstruction. Both informed consent from all subjects and permission from our institution's review board were obtained.

Group A is made up of 603 patients (368 males and 235 females) who have some clinical symptoms (mild or serious) of ureteral obstruction. The results from conventional ultrasonography showed that there was unilateral hydronephrosis and ipsilateral hydroureter in 557 cases (283 on the left and 274 on the right) and no hydronephrosis in the remaining 46 cases. No obstructive lesion could be found by conventional transabdominal ultrasound in all of the 603 cases.

Group B is made up of 174 patients (100 males and 74 females) who have been diagnosed previously with intrahepatic and extrahepatic bile duct ectasia (97 cases) or just extrahepatic bile duct ectasia (77 cases) by conventional ultrasonography. In this group, 117 cases were accompanied with calculus of the gallbladder, 18 cases without, and the rest of the 39 cases had had their gallbladders removed. No obstructive lesion could be visible by conventional transabdominal ultrasound in all of the 174 cases.

### Implementation

The ultrasound scanners employed in our studies were an SSD-280/SSD-1200 scanner (Aloka, Tokyo, Japan) equipped with a 3.5-MHz sector or convex-array probe and a LOGIQ-700 scanner (General Electric, Milwaukee, WI, USA) equipped with a 3 to 8-MHz convex-array probe.

In group A, ultrasonographic examination was performed to detect the ureter with obstructive diseases from different orientations after the patients had been given senna to empty the colon for 12 h, but no obstructive lesion was found. Then, 500 to 1,500 ml warmed normal saline (28°C to 30°C) was slowly injected into the colon of the patients who had no contraindication for the enema, with the bag placed 60 cm above the body and the nozzle inserted 10 to 15 cm into the anus. As to the quantity of normal saline, about 500 ml is sufficient to fill the sigmoid colon fully, 1,000 ml for the descending colon, and 1,500 ml for the transverse colon, ascending colon, and ileocecus (can reach the right pelvic cavity). If too much dejection remained, which can affect acoustic access, the patient was asked to defecate it, and then, normal saline was reinjected into the colon. Through the normal saline fully filled colon, we detected the obstructive lesion along the expanded ureter from proximal to distal till the lesion can be visualized clearly.

In group B, 1,500 ml of normal saline was injected into the patient's colon in the same way as that in group A. With the patients lying down in a supine position, we detected the lesion along the expanded extrahepatic bile duct from proximal to distal through the normal saline-filled transverse colon. If there was some gas in the small intestine and the position of the transverse colon was geometrically lower or higher than that of the obstructive lesions of the bile duct, the patient was asked to lie down in a left lateral recumbent position. Through the flexura hepatica coli and the upper ascending colon, which had moved to the front of the extrahepatic bile duct and were full of normal saline, we detected the obstructive lesion along the expanded extrahepatic bile duct from proximal to distal till the lesion can be visualized clearly. For both groups, the whole process for each case took 5 to 20 min approximately.

## Results

Of the 777 patients, 755 (755/777, about 98%) could hold the normal saline in the colon and succeeded in finishing the examination, and the nature of obstruction in 718 patients (718/755, about 95%) were determined. The results of the ultrasonographic examination after normal saline retention enema in group A and group B are shown in Tables 1 and 2, respectively.

**Table 1:** Results of the ultrasonographic examination after normal saline retention enema in group A

	Total patients	Failing in enema	Succeeding in enema	Missed diagnosis	Confirmed diagnosis	
					Calculus	Stricture
The number of patients	603	13	590	34	533	23
Percentage (%)	-	2.16	97.84	5.76	94.24	
		(13/603)	(590/603)	(34/590)	(556/590)	

**Table 2:** Results of the ultrasonographic examination after normal saline retention enema in group B

	Total patients	Failing in enema	Succeeding in enema	Missed diagnosis	Confirmed diagnosis	
					Calculus	Tumor
The number of patients	174	9	165	3	129	33
Percentage (%)	-	5.17	94.83	1.82	98.18	
		(9/174)	(165/17)	(3/165)	(162/165)	

**Figure 1:**
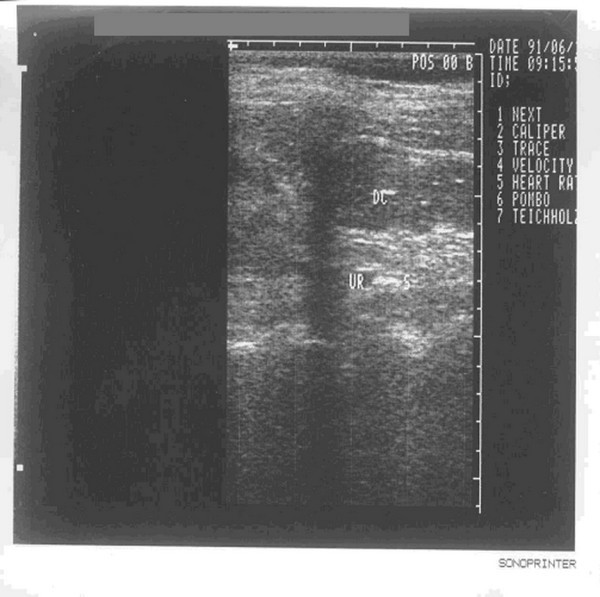
**Ureter calculus**. Seen under transabdominal ultrasound after normal saline retention enema, calculus (indicted by S) of the left ureter (indicted by UR) is visible behind the descending colon (indicated by DC).

**Figure 2:**
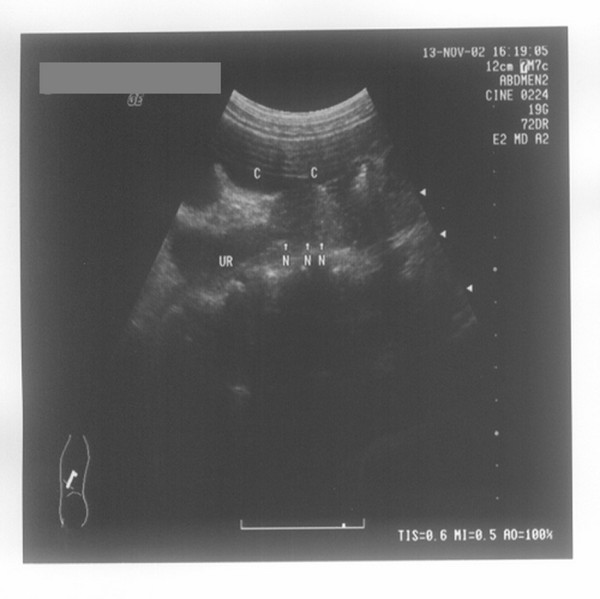
**Ureter stricture**. Seen under transabdominal ultrasound after normal saline retention enema, stricture (indicted by N) of the left ureter (indicted by UR) is visible behind the splenic flexure of the colon and upper descending colon (indicted by C).

**Figure 3:**
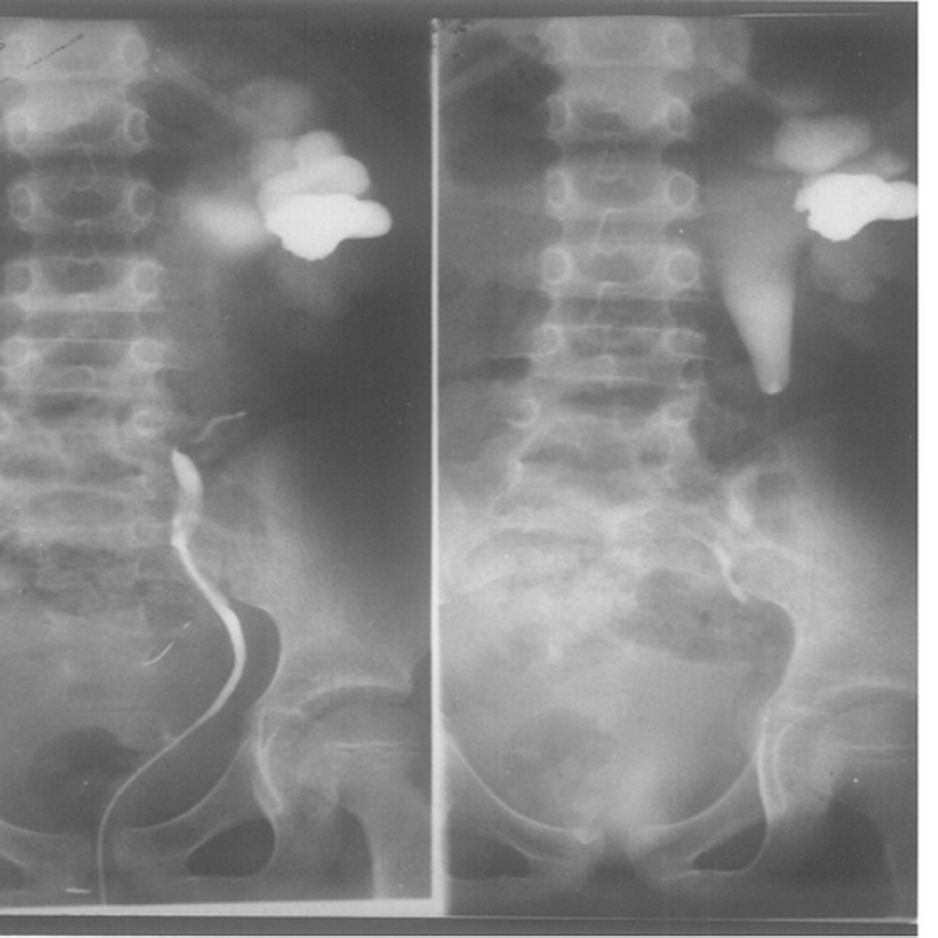
**Ureter stricture seen under X-ray retrograde urethrography and descending urography**. The results from X-ray retrograde urethrography and descending urography of the patient described in Figure 2.

In group A, 590 patients succeeded in the normal saline retention enema, and the rest of the 13 patients (9 males and 4 females) failed to finish the examination because they cannot tolerate the discomfort caused by holding the normal saline in their intestinal canal. The ureteral calculi detected in 533 cases were located behind the normal saline-filled sigmoid colon, descending colon, ascending colon, or ileocecus (see Figure 1). The calculi measured 3 to 20 mm in length, 7.0 mm in average. The inner diameters of the ureter immediately above the calculi were 3 to 15 mm, with an average of 5.7 mm. All of the ureteral strictures detected in 23 cases were located at the pelvi-ureteric junctions. The explicit lumen cannot be seen on the constrictive part, of which the proximal ureter connects to the dilated renal pelvis, due to hydronephrosis and tapers off from the proximal to the distal, presenting the characteristic of 'tapering off'. Meanwhile, no lithiasis or tumor was found (see Figure 2). Figure 3 is from an X-ray retrograde urethrography and descending urography of the patient described in Figure 2, and this patient has been verified by surgical operations. The reason of the 34 cases of missed diagnosis (verified by lith samples excreted out of the body, 13 on the left and 21 on the right) is that these patients are too obese.

**Figure 4:**
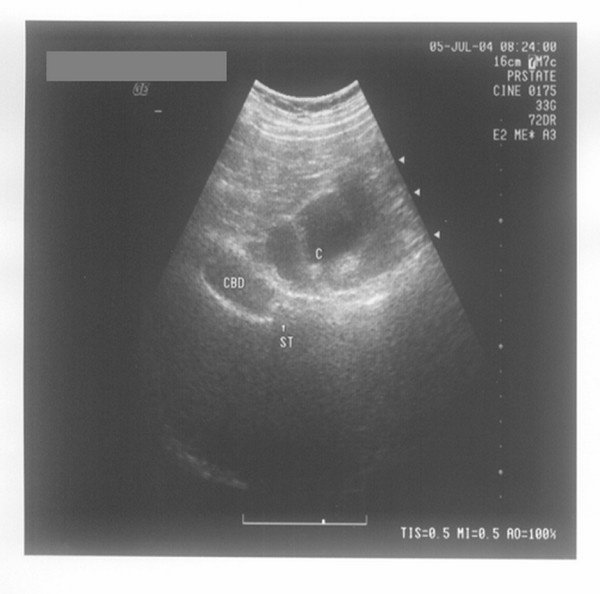
**Extrahepatic bile duct calculi**. Seen under transabdominal ultrasound after normal saline retention enema, sand-like calculi (indicated by ST and the arrows) accumulated at the lower common bile duct (indicated by CBD) are visible behind the transverse colon (indicated by C).

**Figure 5:**
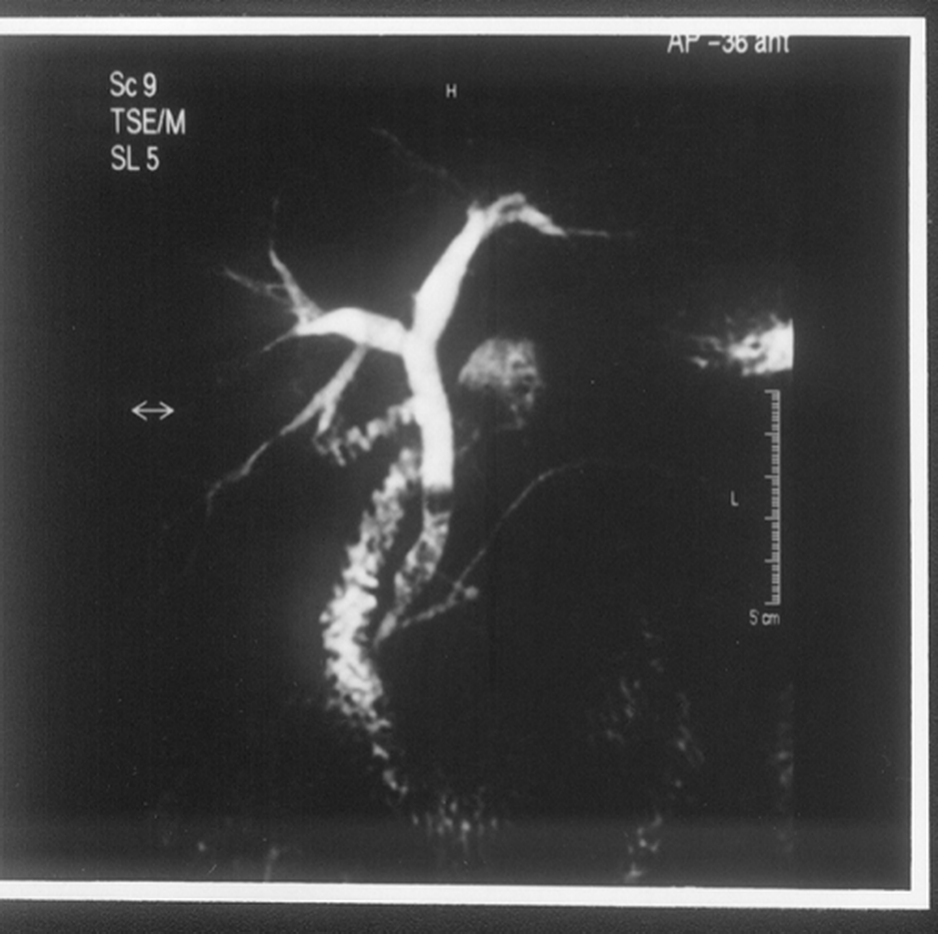
**Extrahepatic bile duct obstruction caused by calculi**. The result from magnetic resonance cholangiopancreatography of the patient described in Figure 4.

**Figure 6:**
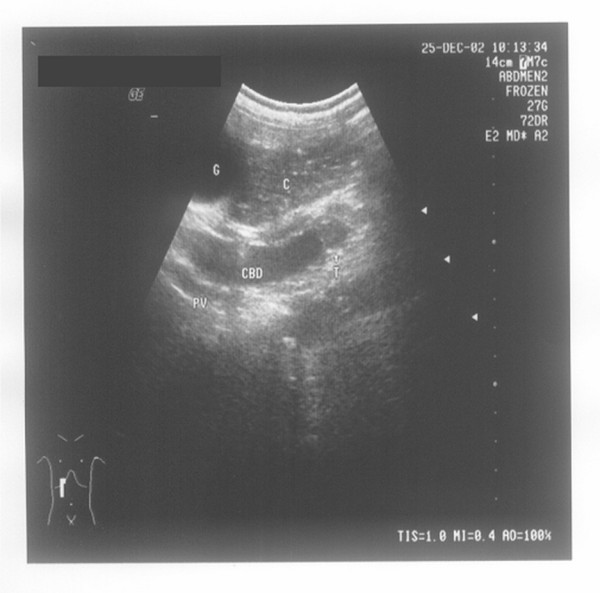
**Extrahepatic bile duct cancer**. Tumors (indicated by T and the arrows) of the common bile duct (indicated by CBD) are visible behind the upper ascending colon (indicated by C). G stands for gallbladder, and PV stands for portal vein.

In group B, 165 patients could tolerate the normal saline retention enema, and the rest of the 9 patients (6 males and 3 females) failed to finish the examination due to the same reason mentioned in group A. Among the calculi detected in the 129 cases, the largest one measures 1.0 × 1.9 cm, while the smallest measures 0.2 × 0.4 cm, and the largest inner diameter of the extrahepatic bile duct is 0.9 to 2.4 cm. The calculi were embedded into the distal common bile duct in 13 patients, including one case who had sand-like calculi accumulated there (Figure 4), which is consistent with the results from the magnetic resonance cholangiopancreatography (Figure 5). Tumors of the middle or distal common bile duct were detected in 33 patients in this study, which were shown on the backside of the upper segment of the colon transversum or ascending colon. The maximum diameter of the common bile duct is 1.2 to 1.3 cm approximately (see Figure 6). There are three cases of missed diagnosis due to severe sound attenuation caused by excessive obesity, the flatulence of the small intestine, the higher position of the transverse colon, and the inapparent left moving of the intestinal duct in left lateral recumbent position. The results of laparotomy showed that all three patients had calculi in the common bile duct, including one case accompanied with purulent cholangitis.

All the ultrasonographic results of the 718 confirmed cases have been verified by follow-up data. The location and nature of the lesions detected by ultrasonography after normal saline retention enema and the results from following up of the patients in group A and group B are shown in Tables 3 and 4, respectively.

**Table 3:** Location and nature of ureteral lesions in group A and the results from the follow-up

Location	Calculi detected by enema diagnosis	Calculi verified by other methods						Stricture detected by enema diagnosis	Stricture verified by other methods	
		Lith excreting of drug	Lith excreting of lithotrity	Lithotrity under X-ray ascending urography	Lith extraction under ureteroscopy	Lithotomy	Total		Shown under X-ray urography	Surgical verification
Above iliac crest plane	-	-	-	-	-	-	-	23 (left 15, right 8)	23	23
Between iliac crest plane and the physiologic secondary stricture of the ureter	300 (left 155, right 145)	83	155	20	35	7	300	-	-	-
Between the physiologic secondary stricture of the ureter and bladder	233 (left 115, right 118)	82	45	10	92	4	233	-	-	-
Total	533	165	200	30	127	11	533	23	23	23

**Table 4:** Location and nature of obstructive lesions in group B and the results from follow-up.

Location	Calculi detected by enema diagnosis	Calculi verified by other methods			Tumors detected by enema diagnosis	Tumors verified surgically
		Shown by ERCP and lith extraction	Surgical verification	Total		
Behind the transverse colon	69	16	53	69	25	25
Behind the hepatic flexure of the colon or ascending colon	60	14	46	60	8	8
Total	129	30	99	129	33	33

All of the pathological results of the tumors from surgical therapy are adenocarcinoma. ERCP, endoscopic retrograde cholangiopancreatography.

## Discussions

Conventional transabdominal ultrasound failed to visualize parts of the ureteral or extrahepatic bile duct covered by bowel gas. In this study, normal saline was employed to fill the colon and replace the bowel gas to form acoustic windows for transabdominal ultrasonography; hence, the structures of the whole ureters and extrahepatic bile duct can be visualized clearly.

The results in Tables 1 and 2 demonstrate that, among those who failed to get the correct diagnosis by conventional transabdominal ultrasound, 94.24% of those with ureter obstruction and 98.18% of those with extrahepatic obstruction can be diagnosed correctly by the method of normal saline retention enema employed in this study.

**Figure 7:**
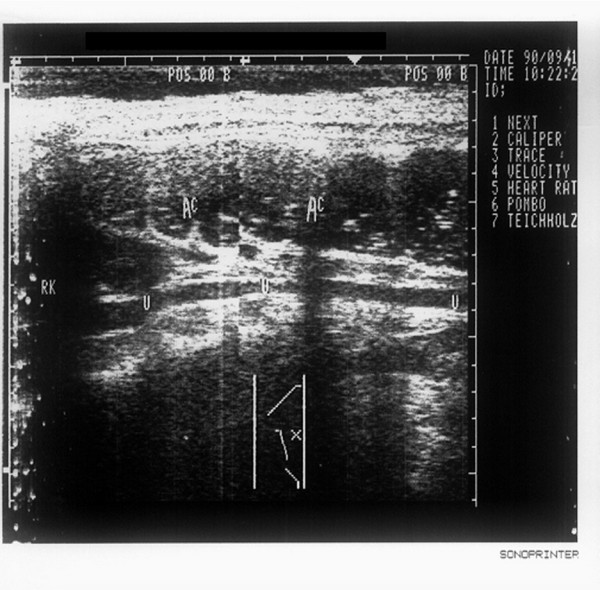
**The whole ureter seen under transabdominal ultrasound after normal saline retention enema**. The right ureter (indicated by U) can be visualized clearly behind the saline-filled ascending colon (indicated by AC). RK stands for right kidney. The calculus in this patient is located in the ureterocystic segmentum posterius, which is not included in the cases studied in this article. No obstructive lesions can be found, and it is taken just as an example to describe the effect of the enema.

It is known from Table 3 that the calculi, which are located in the segment between the iliac crest plane of the ureters and the physiologic secondary stricture of the ureter, and the segment between the physiologic secondary stricture of ureter and the filled bladder, are difficult to detect by conventional sonography. The reason is that these two segments of the ureter are shaded by bowel gas from the frontage and are laterally covered by the pelvis at the backside. The calculi in the second segment are more difficult to visualize owing to its deeper location. Figure 7 (a normal image of the ureter with no obstructive lesion seen under transabdominal ultrasound with normal saline retention enema) shows that, employing the ascending colon which was full of normal saline to gain acoustic access, we can see the expanded right ureter clearly by ultrasonography. Even if there is no hydronephrosis and the expansion of ureter is not obvious, the ureteral calculi can also be clearly visible after the enema (Figure 1). The tapering off characteristic of the stricture in the ureteropelvic junction usually can be visualized by conventional sonography. The reason why the cases listed in this article failed to be visualized by conventional ultrasonography is due to the congenital malrotation of the kidney [10], resulting in the overlap between the left ureteropelvic junction and descending colon or the overlap between the right ureteropelvic junction and ascending colon. After normal saline retention enema, the tapering off characteristic of the left (or right) ureteropelvic junction can be seen just at the backside of the descending colon (or ascending colon) region. Diagnosing a stricture of the ureter according to the tapering off characteristic, we can only estimate the top locality of the stricture, and we are not able to measure the length.

**Figure 8:**
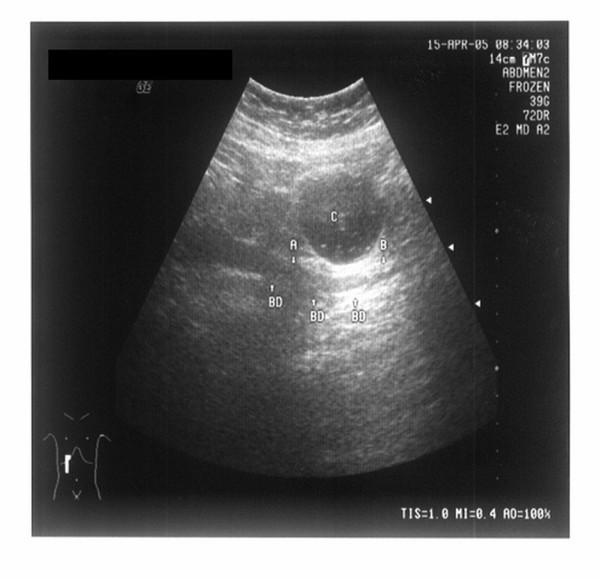
**The whole extrahepatic bile duct seen under transabdominal ultrasound after normal saline retention enema**. The whole compensatory dilated extrahepatic bile duct (indicated by BD and the arrows) is visible behind the transverse colon (indicated by C). Indicated by A and the arrows is the upper bound of the overlapping between the extrahepatic bile duct and transverse colon, and indicated by B and the arrows is the lower bound of the overlapping. This patient was checked after cholecystectomy, and no calculus or cancer was found, which is not included in the cases studied in this article. It is just taken as an example to describe the effect of visualizing the extrahepatic bile duct after the enema.

It is well known that the method of drinking water to fill the stomach had been used to eliminate the effects of stomach air in diagnosing extrahepatic bile diseases by ultrasonography, but it could not solve the problem of the extrahepatic bile duct's being covered by gas in the colon. As the position (higher or lower) of the transverse colon in the body varies from person to person, it has an effect on the ultrasonography results of not only the lower segment of the extrahepatic bile duct but also the upper segment. Figure 8 (a normal image of the extrahepatic bile duct with no obstructive lesion seen under transabdominal ultrasound with normal saline retention enema) shows the overlapping anatomical relationship of the transverse colon (full of normal saline) and the extrahepatic bile duct. The whole compensatory dilated extrahepatic bile duct (the maximum inner diameter is 1.2 cm) is visible after normal saline retention enema (see Figure 8), but only a part of that, i.e., the part above arrow A and below arrow B, can be visualized by conventional ultrasonography. If the obstructive lesions are located in the extrahepatic bile duct behind the transverse colon, the segment A-B of the extrahepatic bile duct, i.e., between arrow A and arrow B, may be difficult to detect with transabdominal ultrasound without normal saline retention enema.

## Conclusions

In normal saline retention enema, the whole colon is fully filled with 1,500 ml of normal saline, expelling the gas from it. After the retention enema, the right and left parts of the colon can give acoustic access to the bilateral ureters, while the colon transversum, hepatic flexure of colon, and the upper ascending colon can give acoustic access to the extrahepatic bile duct, which can help visualize these structures clearly. After normal saline retention enema, the nature of obstruction of the ureter and extrahepatic bile duct in 95% of the patients can be determined by ultrasonography when conventional transabdominal ultrasound fails, and the results are consistent with the follow-up data. This method is easy and convenient to operate with no invasiveness, no pain, and with a reliable result. Most patients, except for a few, can tolerate the retention enema (the success rate of the enema is 97%). It is another method to detect obstructive diseases of the urinary system and extrahepatic bile duct by ultrasonography.

## Abbreviations

CT: computed tomography; MRI: magnetic resonance imaging.

## Competing interests

The authors declare that they have no competing interests.

## Authors' contributions

CT conceived of the study, collected the data, participated in its design and coordination, and helped draft the manuscript. XW participated in its design and helped draft the manuscript. QF drafted and revised the manuscript, and acquired and analyzed the data. ZD carried out the analysis and interpretation of data, and helped revise it critically for important intellectual content. All authors read and approved the final manuscript.

## Authors' information

Chong Tang, MB, works for Xi'an Electric Central Hospital as a medical doctor on sonography and has been studying ultrasonic diagnosis, especially the normal saline retention enema method, for nearly 20 years. Zhensheng Deng, PhD, works for Central South University as a professor.
